# Using automated insulin delivery to address the clinical challenges of glycemic management in people with type 1 diabetes and kidney failure on maintenance hemodialysis

**DOI:** 10.1111/jdi.70141

**Published:** 2025-08-19

**Authors:** Panagiotis Pavlou, Monika Reddy, Parizad Avari, Lalantha Leelarathna, Rachael Tan, Tahseen A. Chowdhury, Rebecca Hyslop, Sufyan Hussain, Thomas Johnston, Janaka Karalliedde

**Affiliations:** ^1^ King's College London London UK; ^2^ Guy's and St Thomas' NHS Foundation Trust London UK; ^3^ Imperial College Healthcare NHS Trust London UK; ^4^ Imperial College London London UK; ^5^ King's College Hospital NHS Foundation Trust London UK; ^6^ Barts Health NHS Trust London UK

**Keywords:** Automated insulin delivery, Diabetic nephropathy, Type 1 diabetes mellitus

## Abstract

Diabetes management in end‐stage kidney disease (ESKD) is complicated by altered insulin pharmacokinetics and glucose metabolism, particularly in the context of hemodialysis (HD). Automated insulin delivery (AID) systems offer dynamic insulin adjustment and may help address these challenges. We present real‐world data from nine individuals (five females) with type 1 diabetes and ESKD on HD, median age 38 years (range 33–50). Over a median follow‐up of 7 months (range 1.5–25), AID use led to significant improvements in glucose time in range (3.9–10 mmol/l) which increased from 39.7% to 59.8% (*P* = 0.001), glucose variability decreased from 39.8% to 33.8% (*P* = 0.01), and HbA1c improved from 78.6 to 56.1 mmol/mol (*P* = 0.003). Time below range fell from 4.0% to 1.4%, but this was not significant (*P* = 0.1). AID was well tolerated and implemented with multidisciplinary support. Our findings highlight the potential of AID to improve glycemic control in this high‐risk population and the need for further studies in this area.

## Graphical abstract

Automated insulin delivery in people with type 1 diabetes and kidney failure on hemodialysis.

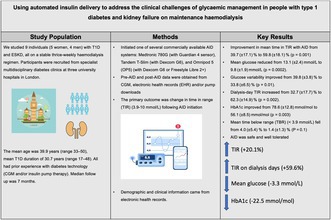

## INTRODUCTION

**Figure 1:**
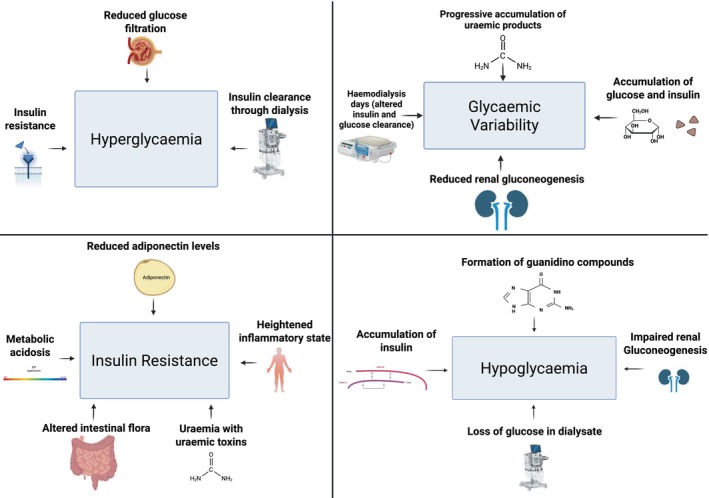
Association of different aspects of ESKD pathophysiology with hypoglycemia, hyperglycemia, glycemic variability, and insulin resistance. Created in https://BioRender.com.

Diabetic kidney disease (DKD) is a leading cause of end‐stage kidney disease (ESKD), affecting up to 40% of people with diabetes^1^, ^2^, ^3^. ESKD complicates diabetes management due to reduced insulin clearance, impaired glucose metabolism, and glycemic variability^4^, ^5^ (see Figure 1). Hemodialysis (HD) further disrupts glucose control by removing insulin and glucose, contributing to both hyper‐ and hypoglycemia^6^. These factors make traditional management unpredictable in this high‐risk group.

Automated insulin delivery (AID) systems, which pair continuous glucose monitoring (CGM) with insulin pumps using adaptive algorithms, offer real‐time insulin adjustments suited to the dynamic physiology of ESKD and HD^7^, ^8^; however, data on their use in this population remain limited.

We previously reported data in four people managed at one hospital with type 1 diabetes (T1D) and ESKD on HD that demonstrated improved time‐in‐range (TIR) over a median 4.5 months with AID^9^. Here, we present further data from an expanded multicenter series of nine further people followed for a median 7 months (see Table 1).

**Table 1:** Patient demographics, baseline and post‐AID CGM parameters

Case	Sex	Age	AID system	CGM data pre‐AID	Follow‐up period (months)	CGM data post‐AID
1	M	47	Omnipod 5/Dexcom G6	Mean (±SD) blood glucose: 12.1 (±4.8)mmol/L [217 (±86.4) mg/dL] Time in target range (TIR): 38% High: 32% Very high: 29% Low: 1% Very low: 0%	5	Mean (±SD) blood glucose: 10.8 (±3.1) mmol/L [194.4 (±55.8) mg/dL] Time in target range (TIR): 46% High: 38% Very high: 16% Low: 0% Very low: 0% BG target: 7.8 mmol/L AIT: 4 h Time in automated mode: 98%
2	F	33	Tandem T‐Slim/Dexcom G6/Control IQ	Mean (±SD) blood glucose: 10.8 (±4.8) mmol/L [194.4 (±86.4) mg/dL] Time in target range (TIR): 53% High: 23% Very high: 23% Low: 1% Very low: 0%	7	Mean (±SD) blood glucose: 10.1 (±4.4) mmol/L [181.8 (±79.2) mg/dL] Time in target range (TIR): 57% High: 24% Very high: 18% Low: 1% Very low: 0% BG target: 6 mmol/L AIT: 5 h Time in automated mode: 95%
3	F	33	Omnipod 5/Decom G6	Mean (±SD) blood glucose: 16.8 (±5.8) mmol/L [302.4 (±104.4) mg/dL] Time in target range (TIR): 16% High: 18% Very high: 66% Low: 0% Very low: 0%	5	Mean (±SD) blood glucose: 11.4 (±3.9) mmol/L [205.2 (±70.2) mg/dL] Time in target range (TIR): 43% High: 33% Very high: 24% Low: 0% Very low: 0% BG target: 8.3 mmol/L AIT: 4 h Time in automated mode: 82%
4	M	35	Medtronic Minimed 780G/Simplera/SmartGuard	Mean (±SD) blood glucose: 10.4 (±4.3) mmol/L [187.2 (±77.4) mg/dL] Time in target range (TIR): 47% High: 29% Very high: 20% Low: 3% Very low: 1%	4	Mean (±SD) blood glucose: 8.3 (±2.7) mmol/L [149.4 (±48.6) mg/dL] Time in target range (TIR): 75% High: 20% Very high: 4% Low: 1% Very low: 0% Time in automated mode: 100% AIT: 2 h
5	F	50	Omnipod 5/Dexcom G6	Mean (±SD) blood glucose: 11.3 (±4.1) mmol/L [203.4 (±73.8) mg/dL] Time in target range (TIR): 39% High: 36% Very high: 24% Low: 1% Very low: 0%	4	Mean (±SD) blood glucose: 7.8 (±2) mmol/L [140.4 (±36) mg/dL] Time in target range (TIR): 84% High: 15% Very high: 0% Low: 1% Very low: 0% Time in automated mode: 100%
6	M	44	Omnipod 5/Freestyle Libre2+	Mean (±SD) blood glucose: 15.1 (±6.4) mmol/L [271.8 (±115.2) mg/dL] Time in target range (TIR): 16% High: 16% Very high: 61% Low: 3% Very low: 5%	5	Mean (±SD) blood glucose: 10.5 (±4)mmol/L [189 (±72) mg/dL] Time in target range (TIR): 48% High: 31% Very high: 18% Low: 2% Very low: 1% Time in automated mode: 98%
7	F	38	Omnipod 5/Freestyle Libre2+	Mean blood glucose^†^: 16.2 mmol/L (291.6 mg/dL) Time in target range (TIR): 28% High: 45% Very high: 25% Low: 2% Very low: 0%	9	Mean blood glucose: 13 mmol/L (234 mg/dL) Time in target range (TIR): 34% High: 23% Very high: 39% Low: 3% Very low: 1% Time in automated mode: 89%
8	F	37	Omnipod 5/Freestyle Libre2+	Mean blood glucose^†^: 12.3 mmol/L (221.4 mg/dL) Time in target range (TIR): 56% High: 28% Very high: 13% Low: 2% Very low: 0%	1.5	Mean blood glucose: 8.8 mmol/L (158.4 mg/dL) Time in target range (TIR): 73% High: 20% Very high: 5% Low: 1% Very low: 1% Time in automated mode: 96%
9	42	M	Medtronic Minimed 780G/Guardian 4/SmartGuard	Mean blood glucose^†^: 13.2 mmol/L (237.6 mg/dL) Time in target range (TIR): 64% High: 10% Very high: 13% Low: 10% Very low: 7%	25	Mean blood glucose: 7.3 mmol/L (131.4 mg/dL) Time in target range (TIR): 78% High: 16% Very high: 5% Low: 1% Very low: 0% AIT: 3 h

AID, automated insulin delivery; AIT, active insulin time; CGM, continuous glucose monitor; SD, standard deviation.

^†^ Blood glucose SD was not available for these patients.

## MATERIALS AND METHODS

We studied nine individuals (five women, four men) with T1D and ESKD, all on a stable thrice‐weekly HD regimen. Participants were recruited from specialist multidisciplinary diabetes clinics at three university hospitals in London. The mean age was 39.9 years (range 33–50), with a mean T1D duration of 30.7 years (range 17–48). All had prior experience with diabetes technology (CGM and/or insulin pump therapy).

Participants initiated one of several commercially available AID systems, chosen based on personal preference and center expertise. These included Medtronic 780G (with Guardian 4 sensor), Tandem T‐Slim (with Dexcom G6), and Omnipod 5 (OP5) (with Dexcom G6 or Freestyle Libre 2+). Pre‐AID data were obtained from CGM, electronic health records (EHR) and/or pump downloads for at least 14 days, and post‐AID data were collected after a minimum of 8 weeks. Demographic and clinical information came from electronic health records.

To account for altered insulin clearance with ESKD and HD, conservative AID settings were used, with glucose targets of 6.1–6.8 mmol/L and active insulin time (AIT) of 4 h or more. With the same clinical rationale, adjustments to basal rates for OP5 and insulin sensitivity (ISF) for Tandem‐T‐slim were made to account for algorithm particularities. All participants had ophthalmologic review prior to AID to exclude active sight‐threatening retinopathy.

The primary outcome was change in time‐in‐range (TIR; 3.9–10 mmol/L). Secondary outcomes included time below range (TBR), time in very high range (TVH) (≥14 mmol/L), glucose variability (GV), mean glucose, HbA1c, total daily insulin (TDI), weight, and dialysis‐day‐specific metrics. Paired *t*‐tests evaluated statistical significance.

Time in range is defined as the percentage of time spent at CGM glucose levels between 3.9 and 10 mmol/L, time below range as the percentage of time spent at <3.9 mmol/L, and time in very high range as the percentage of time spent at glucose levels >13.9 mmol/L^10^. Standard of care goals for CGM metrics include >70% TIR and <4% TBR^10^. Glucose variability is defined as the coefficient of variation, calculated by dividing the standard deviation of sensor glucose values by the mean of sensor glucose values during the observation period^7^. It is associated with the risk of hypoglycemia, development of diabetic retinopathy, and the risk of cardiovascular disease^11^, ^12^, ^13^.

## RESULTS

For further results and information on all the cases, please see Table 2.

**Table 2:** Changes in CGM parameters, HbA1c and weight post‐AID

	Mean pre‐AID (±SD)	Mean post‐AID (±SD)	Difference post‐AID (Δ)	*P*‐value	Number of patients (*N*)
TIR (%)	39.7 (±17.1)	59.8 (±18.1)	20.1	0.001	9
Time in very high range (%)	30.4 (±19.5)	14.3 (±12.3)	−16.1	0.01	9
TBR (%)	4 (±5.4)	1.4 (±1.3)	−2.6	0.1	9
Mean BG (mmol/L)	13.1 (±2.4)	9.8 (±1.9)	−3.3	0.0002	9
GV (%)^†^	39.8 (±3.8)	33.8 (±6.5)	−6	0.01	6
TDI (IU)^‡^	35.8 (±12.2)	29.6 (±7.1)	−6.2	0.05	6
Hemodialysis day TIR (%)^§^	32.7 (±17.7)	62.3 (±14.9)	29.6	0.002	6
Hemodialysis day TDI (IU)^¶^	27.3 (±7.9)	26.9 (±5.8)	−0.32	0.43	4
HbA1c (mmol/mol)^††^	78.6 (±12.8)	56.1 (±8.5)	−22.5	0.003	8
Weight^‡‡^	74.3 (±19.6)	71.8 (±15.9)	−2.5	0.23	5

BG, blood glucose; GV, glucose variability; HbA1c, glycated hemoglobin A1c; TDI, total daily insulin; TIR, time in range, time below range.

^†^ GV data were not available for three patients.

^‡^ TDI data were not available for three patients.

^§^ Hemodialysis day TIR data were not available for three patients.

^¶^ Hemodialysis day TDI data were not available for three patients.

^††^ HbA1c data at suitable time points were not available for one patient.

^‡‡^ Weight data were not available for three patients.

### Case 1

A 47‐year‐old male with T1D since age 14, ESKD on HD for 2 years, hypertension, and HIV. Previously on multiple daily injections (MDI), he commenced OP5 with Dexcom G6 5 months ago. Pre‐AID, mean TIR was 38%, Very high 29%, High 32%, GV 39%. Post‐AID, mean TIR improved to 46%, Very high to 16%, GV to 29%, and dialysis‐day TIR rose from 25% to 48.8%. Time in automated mode was 98%; AIT 4 h; blood glucose (BG) target 7.8 mmol/L.

### Case 2

A 33‐year‐old female with T1D for 17 years, ESKD, failed simultaneous pancreas–kidney (SPK) transplant, retinopathy, neuropathy, and hypertension. Received HD for 2 years and switched from manual pump therapy to Tandem T‐Slim/Control IQ. Pre‐AID CGM data showed nocturnal hyperglycemia. Post‐AID, Very High reduced from 23% to 18%. Time in automated mode was 95%; BG target 6 mmol/L; AIT 5 h.

### Case 3

A 33‐year‐old female with a 24‐year T1D history, ESKD, retinopathy, neuropathy, gastroparesis, hypertension, awaiting SPK. On HD for 1 year, transitioned from MDI to OP5 with Dexcom G6. Pre‐AID: TIR 16%, Very High 66%. Post‐AID: TIR 43%, Very high 24%, dialysis‐day TIR from 19% to 45.3%. Time in auto mode 82%; BG target 8.3 mmol/L; AIT 4 h.

### Case 4

A 35‐year‐old male with T1D for 32 years, ESKD post‐failed kidney transplant, retinopathy, neuropathy, hypertension, and heart failure (HF). On HD for 3 years; transitioned from manual pump therapy to Medtronic 780G/SmartGuard AID. Mean TIR increased from 47% to 75%; Very High from 20% to 4%; GV from 41.3% to 32.5%. Dialysis‐day TIR rose from 55.5% to 82.6%. Time in auto mode 100%; AIT 2 h.

### Case 5

A 50‐year‐old female with T1D since infancy, ESKD, antiphospholipid syndrome, retinopathy, peripheral vascular disease with lower limb amputation, and HF switched from MDI to OP5 with Dexcom G6. Pre‐AID TIR 62%; post‐AID 67%. Dialysis‐day TIR improved from 36% to 76.6%. Time in auto mode 100%.

### Case 6

A 44‐year‐old male with T1D since age 4, ESKD, impaired hypoglycemia awareness, severe hypoglycemia, retinopathy, autonomic neuropathy, gastroparesis, and HF. Switched from MDI to OP5 with Freestyle Libre 2 plus. TIR improved from 16% to 48%; dialysis‐day TIR from 9.7% to 62.5%; TBR reduced from 8% to 3%. Time in auto mode 91%.

### Case 7

A 38‐year‐old female with T1D since age 21, retinopathy, neuropathy, and ESKD on HD since 2022. Previously on MDI, started OP5 with Freestyle Libre 2+ and basal insulin. TIR improved from 28% to 36%; HbA1c dropped from 76 to 67 mmol/mol. Time in auto mode 89%.

### Case 8

A 37‐year‐old female with T1D since age 13, retinopathy, and ESKD on HD since 2022. Switched from MDI to OP5 with Freestyle Libre 2+. TIR rose from 56% to 64%; TBR reduced from 8% to 4%. Time in auto mode 96%.

### Case 9

A 42‐year‐old male with long‐standing poorly controlled T1D, ESKD on HD, retinopathy, and coronary artery disease. Previously on MDI. Started Medtronic 780G/SmartGuard in March 2023. Post‐AID, TIR increased to 78%; TBR <4%, with reduction in hypoglycemic episodes. AIT was set for him at 3 h.

In this case series of 9 people we observed over a median follow‐up of 7 months, a significant improvement in mean time in TIR (± standard deviation [SD]) from 39.7 (±17.1) to 59.8 (±18.1) % (*P* = 0.001). Time in the very high range decreased from 30.4 (±19.5) % to 14.3 (±12.3) % (*P* = 0.01), and mean glucose reduced from 13.1 (±2.4) mmol/L to 9.8 (±1.9) mmol/L (*P* = 0.0002). Glucose variability (GV) fell from 39.8 (±3.8) % to 33.8 (±6.5) % (*P* = 0.01). On dialysis days, mean TIR increased from 32.7 (±17.7) to 62.3 (±14.9) % (*P* = 0.002). HbA1c improved significantly, from 78.6 (±12.8) mmol/mol to 56.1 (±8.5) mmol/mol (*P* = 0.003). Although mean time below range (TBR) fell from 4.0 (±5.4) % to 1.4 (±1.3) %, this was not statistically significant (*P* = 0.1). Decrease in TDI dose was observed from 35.8 (±12.2) IU to 29.6 (±7.1) IU (*P* = 0.05), while changes in dialysis‐day TDI (from 27.3 [±7.9] IU to 26.9 [±5.8] IU; *P* = 0.43) and weight (from 74.3 [±19.6] kg to 71.8 [±15.9] kg; *P* = 0.23) were not significant. No episodes of diabetic ketoacidosis or severe hypoglycemia requiring third‐party assistance were reported during follow‐up.

## DISCUSSION

This multicenter case series study is the first to demonstrate statistically significant improvements in multiple glycemic outcomes with AID in people with T1D and ESKD on HD. Over a median follow‐up of 7 months, we observed clinically meaningful significant improvements in TIR, GV, HbA1c, mean glucose, and dialysis‐day specific glycemic outcomes. These results build on earlier work with a smaller cohort and shorter follow‐up^9^, adding strength and relevance to our findings through inclusion of patients from diverse ethnic backgrounds with complex comorbidities. Despite its retrospective nature and small sample size, our study supports AID as a feasible and safe therapy in this high‐risk group where there is sparse clinical data or evidence. In our opinion, pragmatic, conservative AID settings and multidisciplinary clinical review and support were key to the safety and effectiveness of delivering AID. Our results demonstrate that AID shows promise for improving glucose control, reducing the burden of glycemic variability and extremes of glycemia in people with T1D and ESKD on HD. Combined with limited prior studies in type 2 diabetes and of short duration^8^, ^14^, our findings highlight the need for larger scale research in this area.

## FUNDING

None.

## DISCLOSURE

LL reports personal fees from Abbott Diabetes Care, Dexcom, Insulet, Roche, Medtronic, Novo Nordisk, and Sanofi. SH has served on the advisory board for Tandem, Dexcom, and Medtronic; undertaken non‐promotional educational and/or consultancy work for Abbott UK, Insulet, Dexcom, and Roche; received an unrestricted educational research grant from Abbott UK and an investigator‐initiated research grant from Insulet. SH reports honoraria from Medtronic, Abbott, Dexcom, Insulet, and Tandem. The remainder of the authors do not have anything to disclose.

Approval of the research protocol: N/A.

Informed consent: Informed consent was obtained from all patients involved.

Registry and the registration no. of the study/trial: N/A.

Animal studies: N/A.

## AUTHOR CONTRIBUTIONS

J.K. and P.P. conceived the concept of the study. P.P. and J.K. wrote the manuscript; P.P. performed the literature investigation, collected and collated the data, prepared the figures and tables, and performed the statistical analysis. J.K., P.A., L.L., T.C., and S.H. critically reviewed and edited the manuscript. P.P., P.A., L.L., T.C., R.T., T.J., and R.H. contributed to data collection. J.K. supervised the study. All authors read and approved the final manuscript.

## Data Availability

The data that support the findings of this study are available from the corresponding author upon reasonable request.
